# Coronary artery disease is associated with an altered gut microbiome composition

**DOI:** 10.1371/journal.pone.0227147

**Published:** 2020-01-29

**Authors:** Takumi Toya, Michel T. Corban, Eric Marrietta, Irina E. Horwath, Lilach O. Lerman, Joseph A. Murray, Amir Lerman

**Affiliations:** 1 Department of Cardiovascular Medicine, Mayo Clinic, Rochester, MN, United States of America; 2 Department of Cardiology, National Defense Medical College, Tokorozawa, Saitama, Japan; 3 Department of Gastroenterology, Mayo Clinic, Rochester, MN, United States of America; 4 Division of Nephrology and Hypertension, Mayo Clinic, Rochester, MN, United States of America; University of Bologna, ITALY

## Abstract

Alteration of gut microbiome composition has been linked to cardiovascular diseases. To identify specific bacterial communities associated with coronary artery diseases (CAD), we conducted a case-control study with 53 advanced CAD patients and 53 age-, sex-, race-, and BMI-matched controls. V3-V5 regions of the 16S rDNA from the fecal gut material were analyzed to compare the gut microbiome composition between CAD patients and controls. The alpha diversity, including Chao-1, Shannon-index, and the number of observed taxonomy units were significantly decreased in CAD patients indicating, decreased richness and evenness of gut microbiome. Among 23 different abundant taxa at the genus level, 12 taxa belonged to *Lachnospiraceae* family, which are known to produce butyrate. Further, we identified five taxa which showed more than two log-fold changes with maximum proportion >0.002, including *Ruminococcus gnavus*, *Lachnospiraceae anaerosporobacter*, *Lachnospiraceae NK4B4* group, *Lachnospiraceae UCG-004*, and *Ruminococcus gauvreauii*. After adjustment for coronary risk factors (diabetes mellitus and dyslipidemia), decreased relative abundance of *Lachnospiraceae NK4B4* group and *Ruminococcus Gauvreauii* and increased relative abundance of *Ruminococcus gnavus* were associated with the presence of advanced CAD. The observed differences in taxa between CAD patients and controls in this study may provide insight into the link between the gut microbiome and CAD.

## Introduction

More than 2000 species of commensal bacterial organisms reside in and on our body, providing us with the metabolic benefit of additional genes and activities [1]. The gut microbiome is by far the greatest mass of our microbiota and has developed many complex networks with the hosts, and thus dysbiosis of the gut microbiome has been implicated to a wide variety of diseases and conditions such as inflammatory bowel disease, metabolic diseases, malignancies, psychiatric disorders, immune disorders, and cardiovascular diseases [2]. Based on the understanding of the physiological roles of the gut microbiome, many gut microbiome-derived metabolites observed in the blood and urine could be used to identify patients at risk [3]. Gut microbiome uses nutrients such as lecithin, choline, betaine, and carnitine as a carbon source and wastes trimethylamine which is oxidized by hepatic enzymes, particularly flavin monooxygenase 3, to form trimethylamine-N-oxide (TMAO). Plasma level of TMAO is strongly correlated with the severity of coronary artery disease (CAD), suggestive that gut microbiome may be manipulated to reduce cardiovascular disease burden [4]. In fact, some prebiotic and probiotic treatment successfully showed cardiovascular benefit in mice [5–8]. However, the application of experimental animal data to CAD in human requires caution and further evaluation, given major differences in gut microbiome between mice and humans. In addition, many confounding factors further influence gut microbiome composition in human, including sex, race, age, and lifestyles such as diet and obesity [9–13]. To investigate the association between specific gut microbes on the development of CAD, these confounding factors should be accounted for in any observed alteration; however, human gut microbiome data that demonstrates a clear association between the carriage or absence of particular microbes and human CAD is still weak. Therefore, we performed the current study to investigate the composition and to infer functional differences in the gut microbiome of patients with CAD using 16S ribosomal DNA (rDNA) microbiome analysis.

## Materials and methods

### Study population

**Fig 1:**
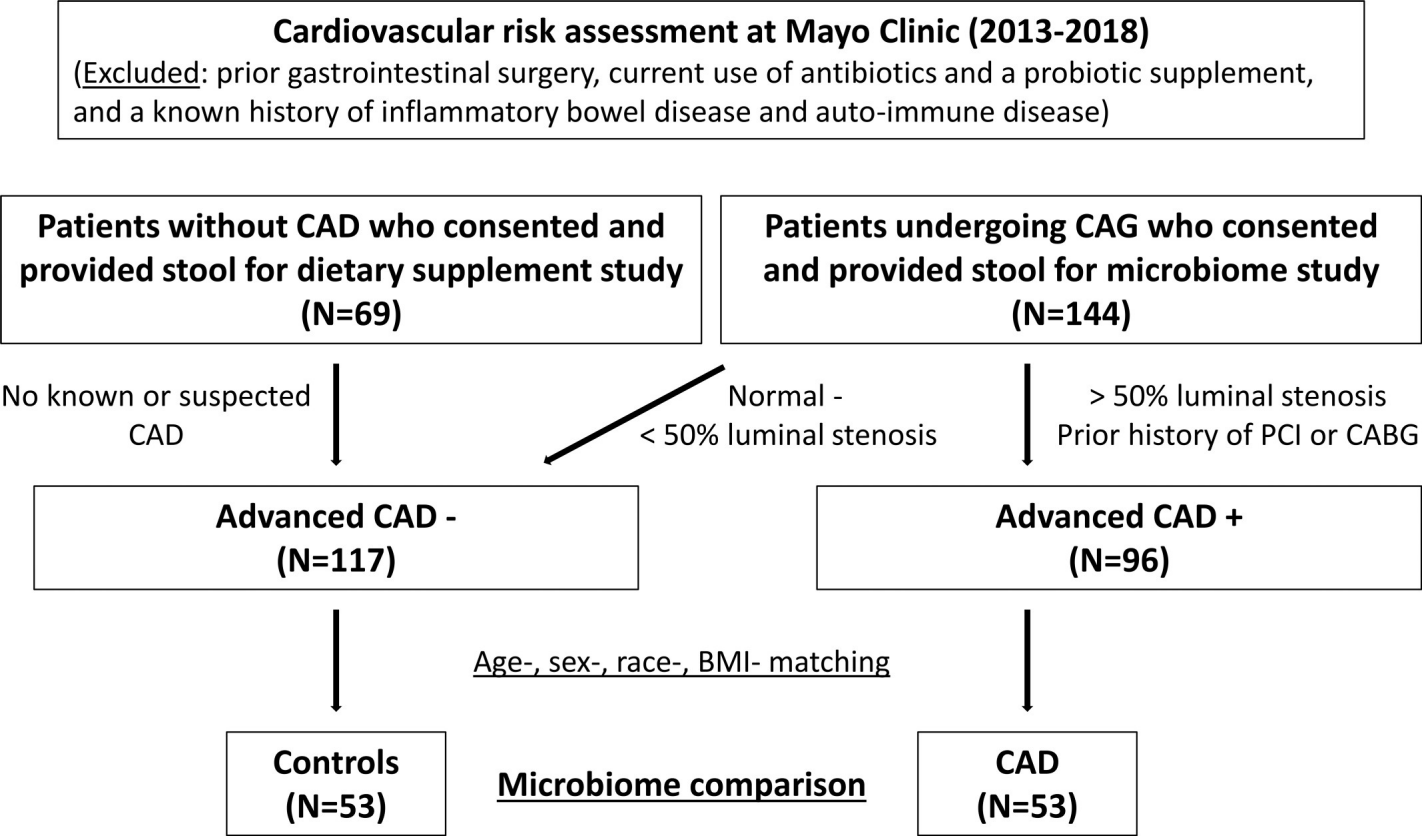
Flowchart explaining CAD patients and controls recruited for the study. We enrolled 213 participants (96 patients with advanced CAD and 117 patients without advanced CAD) in the microbiome study. Fifty-three advanced CAD patients and age-/sex-/race-/BMI- matched participants without advanced CAD were ultimately compared.

In this single-center, case-control study, we enrolled 213 patients who underwent cardiovascular risk assessment at Mayo Clinic between December 2013 and November 2018 (Fig 1). One hundred forty-four patients out of 213 patients were consecutive patients who consented to participate and provided stool for microbiome study at the time of clinically indicated coronary angiography for the assessment of chest pain and/or dyspnea. Significant coronary artery disease (CAD) defined as more than 50% of luminal stenosis was detected in 96 patients. The remaining 69 patients, without known or suspected CAD based on clinical history, noninvasive stress testing, and coronary imaging studies including coronary computed tomography and/or coronary angiography, were those enrolled in a different study investigating the effects of a dietary supplement on endothelial function between February 2015 and February 2017. Only the baseline microbiome samples were included in the final analysis. Subjects with prior gastrointestinal surgery including colectomy, ileectomy, and gastrectomy, current administration of antibiotics and a probiotic supplement, and a known history of inflammatory bowel disease and auto-immune diseases, were excluded.

To investigate the compositional change of microbiome associated with CAD, we created age-, sex-, race-, and BMI-matched control and CAD group on one to one basis, resulting in a total of 53 controls and 53 patients with advanced CAD.

The study was conducted in accordance with the guidelines of the Declaration of Helsinki and was approved by the Mayo Clinic Institutional Review Board. All patients provided written informed consent for participation in the current protocol.

### Clinical assessment

Clinical history, laboratory data, and the current medications were collected from detailed chart review. Data were collected on the following parameters: 1) sex, age, body mass index (BMI), and traditional coronary vascular disease risk factors (smoking status and obesity [BMI >30 kg/m^2^), 2) dyslipidemia, defined by a documented history of hyperlipidemia, treatment with lipid-lowering therapy, a low-density lipoprotein cholesterol (LDL-C) above target (<130 mg/dL for low risk patients, <100 mg/dL for moderate-high risk patients, <70 mg/dL for very high risk, and <55 mg/dL for extreme high risk patients based on 10-year atherosclerotic cardiovascular disease risk), high density lipoprotein cholesterol <40 mg/dL in men or <50 mg/dL in women, or triglyceride >150 mg/dL, 3) type 2 diabetes mellitus, defined as a documented history or treatment of type 2 diabetes, 4) hypertension, defined as a documented history of or treatment for hypertension, and 5) CAD, defined as a documented history of percutaneous coronary intervention or coronary artery bypass grafting for significant coronary artery stenoses, or more than 50% of luminal stenosis in any coronary arteries diagnosed by coronary angiography.

### Assessment of gut microbiome composition

To investigate the compositional change of microbiome associated with CAD, 16S rDNA of collected stool samples were analyzed using methods described previously [14]. Participants were provided with stool collection kits (Fisher Scientific Inc., Pittsburgh, PA, USA). If patients were unable to give the stool samples during their stay at Mayo Clinic, they were instructed to collect the sample at home and ship it through overnight FedEx delivery. Samples were frozen at -70 °C within 24 hours of receipt. Microbial DNA was extracted from the fecal material of each sample using the Mobio PowerSoil Kit (MoBio Laboratories, Carlsbad, CA, USA) as per the manufacturer's instruction with a bead-beating step. Sequencing of the V3-V5 region of the 16S data was performed and raw 16S data were processed by IM-TORNADO to form operational taxonomic units (OTU) at 97% similarity level [15]. Alpha-diversity measures were calculated based on the rarefied OTU counts. Number of observed OTU and Chao-1 index indicates microbial richness, which measures the number of taxa in each sample. Shannon index indicates microbial evenness, which measures the relative number of taxa in samples accounting for the number of times each taxon was observed in a sample.

### Statistical analysis

Continuous variables distributed normally were expressed as the mean ± standard deviation (SD), and those with a skewed distribution were expressed as the median with interquartile range (IQR). Categorical variables were expressed as frequency (percentage). Patients were divided into two groups—those with advanced CAD (CAD) and those without advanced CAD (Controls). To compare variables between groups, we performed an unpaired t-test for normally distributed continuous variables, a Mann-Whitney U test for non-normally distributed variables, and χ^2^ test (and Fisher exact test) for categorical variables. A P-value <0.05 was considered statistically significant. Differential abundance analysis was performed using the Wilcoxon rank-sum test at genus levels. False discovery rate control based on the Benjamini-Hochberg procedure was used to correct for multiple testing. Univariate and multivariate logistic regression analysis was performed to investigate the effects of variables including conventional coronary risk factors and the compositional change of gut microbes with the cut-offs of two log-fold changes and maximum proportion >0.002 on CAD development. All statistical analyses were performed using JMP Pro software (SAS Institute, Inc., Cary, NC, USA).

## Results

### Baseline characteristics

Comparison of baseline characteristics between patients with advanced CAD and those without advanced CAD in the whole population is summarized in S1 Table. Patients with advanced CAD were significantly older and more likely to have traditional cardiovascular risk factors. Therefore, we created age, sex, race, and BMI matched controls and CAD patients. Table 1 outlines the baseline characteristics of the study population, including controls and CAD patients. CAD patients were significantly more likely to have traditional cardiovascular risk factors, such as diabetes mellitus and dyslipidemia even after age, sex, race, and BMI matching. CAD patients were also more likely to be treated with aspirin, statin, vasodilators, and antidiabetic medications.

**Table 1:** Baseline characteristics.

	Controls N = 53	CAD patients N = 53	*P* Value
Age, years	61.6±10.0	64.1±8.6	0.17
Sex, n (%)			
Male	30 (56.6)	32 (60.4)	0.69
Female	23 (43.4)	21 (39.6)	
Race, n (%)			
Caucasian	53 (100.0)	53 (100.0)	
Non-Caucasian	0 (0.0)	0 (0.0)	
Comorbidities, n (%)			
Hypertension	26 (49.1)	25 (47.2)	0.85
Diabetes Mellitus	3 (5.7)	15 (28.3)	0.002
Dyslipidemia	23 (45.1)	39 (73.6)	0.003
Chronic kidney disease	7 (13.2)	7 (13.2)	
Coronary artery disease	0 (0.0)	53 (100.0)	<0.0001
Smoking			
Current	4 (7.6)	5 (9.4)	0.62
Former	19 (35.9)	23 (43.4)	
Never	30 (56.6)	25 (47.2)	
Laboratory data			
LDL-C, mg/dL	118.2±42.5	89.0±32.3	0.002
HDL-C, mg/dL	54 (46–63)	46 (39–59)	0.028
Triglyceride, mg/dL	107 (75–148)	123 (97–163)	0.010
FPG, mg/dL	97 (91–103)	105 (98–140)	0.001
Creatinine, mg/dL	0.97±0.23	0.99±0.20	0.72
BMI, kg/m^2^	29.1±5.7	29.4±5.9	0.76
Systolic BP, mmHg	123.9±18.7	131.8±18.1	0.028
Diastolic BP, mmHg	74.7±9.9	71.9±10.7	0.17
Medications, n (%)			
Aspirin	30 (56.6)	43 (81.1)	0.006
Statins	21 (39.6)	39 (73.6)	0.0004
Long-acting nitrate	6 (11.3)	24 (45.3)	0.0001
Antihypertensive	35 (66.0)	39 (73.6)	0.40
Antidiabetic	3 (5.7)	13 (24.5)	0.007
Proton-pump inhibitor	12 (22.6)	8 (15.1)	0.32
Multi-vitamins	20 (37.7)	16 (30.2)	0.41
Alcohol consumption, drinks/week	1 (0–4)	1.5 (0–2)	0.48

CAD, coronary artery disease; LDL-C, low-density lipoprotein cholesterol; HDL-C, high-density lipoprotein cholesterol; FPG, fasting plasma glucose; BMI, body mass index; BP, blood pressure.

Excluded patients from the microbiome analysis to create age-, sex-, BMI-matched cohorts were younger and had less comorbidity than included patients (S2 Table).

### Comparison of gut microbiome composition

**Fig 2:**
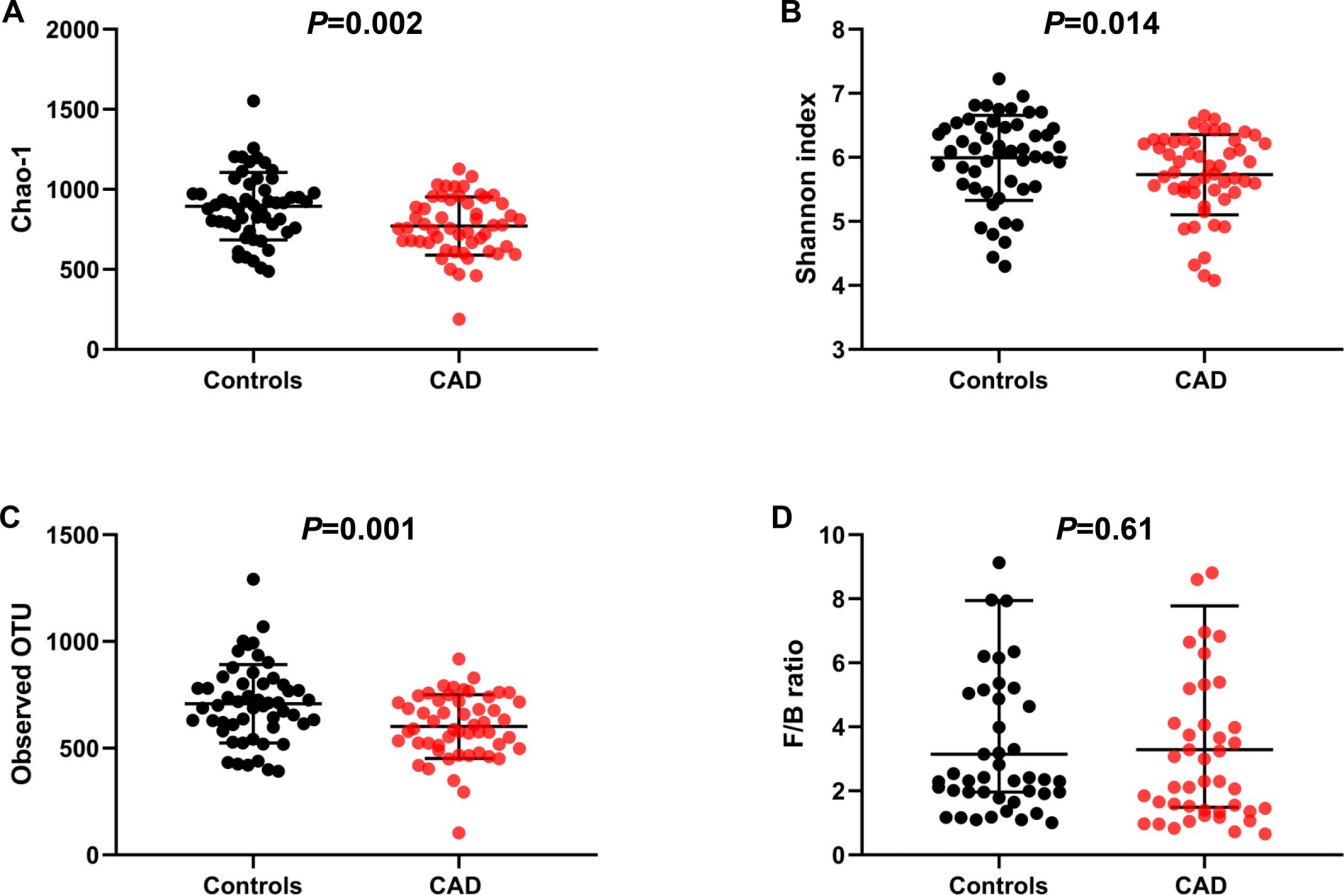
Gut microbial change in patients with or without advanced CAD. **(**A) Chao-1. (B) Shannon-index. (C) Observed OTU. (D) The ratio of *Fermicutes* to *Bacteroidetes*.

**Fig 3:**
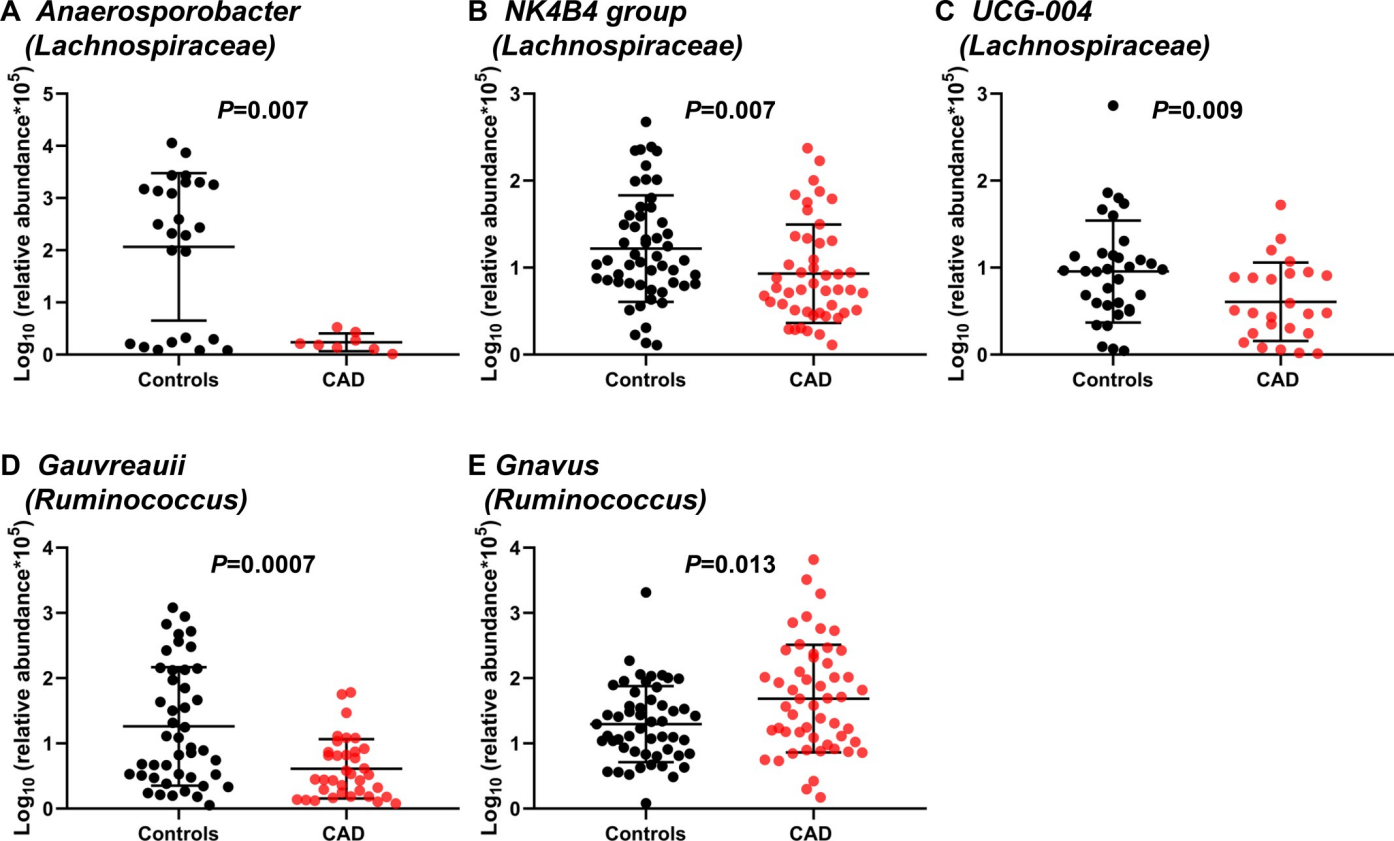
Comparison of relative abundance of gut microbes between patients with and without advanced CAD. (A) *Lachnospiraceae Anaerosporobacter*. (B) *Lachnospiraceae NK4B4*. (C) *Lachnospiraceae UCG-004*. (D) *Ruminococcus Gauvreauii*. (E) *Ruminococcus Gnavus*.

We compared the gut microbiome profile using 16S rDNA gene sequencing in fecal samples between CAD patients and controls. The α diversity measures, which included Chao-1 and number of observed OTU indicating richness and Shannon index indicating evenness, were significantly decreased in CAD patients than controls (*P* = 0.002, *P* = 0.001, and *P* = 0.014, respectively) (Fig 2A–2C). The ratio of *Firmicutes to Bacteroidetes* (two major bacterial phyla consisting of gut microbiome), which has been used as a simple marker of dysbiosis, was not different between the two groups (Fig 2D). A comprehensive comparison between CAD patients and controls in terms of the relative abundance at the genus level was performed using a conservative Wilcoxon rank-sum test. Among 23 differently abundant taxa (*P* <0.05), 12 taxa belonged to *Lachnospiraceae* family (Table 2). After correction for multiple comparisons, only three taxa including, *Lachnospiraceae Anaerosporobacter*, *Lachnospiraceae K4B4* group, and *Ruminococcus Gauvreauii* group, showed significantly lower abundance in CAD patients compared to controls at a false discovery rate of 5% (*Q* = 0.017, *Q* = 0.028, and *Q* = 0.017, respectively) (Table 2). Using maximum proportion >0.002 and two log-fold changes as cut-offs, we observed that CAD patients showed enrichment of *Ruminococcus Gnavus*; whereas controls showed enrichment of *Lachnospiraceae Anaerosporobacter*, *Lachnospiraceae NK4B4* group, *Lachnospiraceae UCG-004*, and *Ruminococcus Gauvreauii* group (Table 2 and Fig 3).

**Table 2:** Differentially abundant taxa between control and CAD patient samples at genus level.

*Phylum/* *Class*	*Order*	*Family*	*Genus*	*P* value	*Q* value	Controls (N = 53) Mean	CAD (N = 53) Mean	Log2 fold change
***Actinobacteria***	* *	* *	* *					
*Coriobacteriia*	*Coriobacteriales*	*Coriobacteriaceae*	*Enorma*	0.004	0.14	1.23E-04	1.23E-04	0.00
***Bacteroidetes***								
*Bacteroidia*	*Bacteroidales*	*Porphyromonadaceae*	*Porphyromonas*	0.032	0.35	1.19E-04	1.38E-04	0.21
		*Prevotellaceae*	*Prevotella_7*	0.041	0.42	1.04E-03	1.43E-03	0.46
***Firmicutes***								
*Clostridia*	*Clostridiales*	*Christensenellaceae*	*Christensenellaceae_R-7*	0.047	0.37	1.82E-02	1.14E-02	-0.67
		*Family_XIII*	*Family_XIII_UCG-001*	0.011	0.21	4.13E-04	2.35E-04	-0.81
		*Lachnospiraceae*	*Agathobacter*	0.013	0.22	2.44E-02	2.46E-02	0.01
		*Lachnospiraceae*	*Anaerosporobacter*	<0.0001	*0*.*017*	6.72E-03	2.78E-06	-11.24
		*Lachnospiraceae*	*Coprococcus_3*	0.029	0.39	2.90E-03	1.92E-03	-0.59
		*Lachnospiraceae*	*Eisenbergiella*	0.0026	0.11	1.76E-03	1.28E-03	-0.46
		*Lachnospiraceae*	*Fusicatenibacter*	0.032	0.33	1.33E-02	7.22E-03	-0.88
		*Lachnospiraceae*	*Lachnospiraceae_ND3007*	0.0073	0.16	1.17E-03	7.80E-04	-0.58
		*Lachnospiraceae*	*Lachnospiraceae_NK4B4*	0.0005	*0*.*028*	4.75E-04	2.08E-04	-1.19
		*Lachnospiraceae*	*Lachnospiraceae_UCG-004*	0.007	0.17	2.29E-04	3.45E-05	-2.73
		*Lachnospiraceae*	*[Eubacterium]_hallii*	0.042	0.40	2.26E-02	1.64E-02	-0.46
		*Lachnospiraceae*	*[Ruminococcus]_gauvreauii*	0.0002	*0*.*017*	1.08E-03	5.50E-05	-4.30
		*Lachnospiraceae*	*[Ruminococcus]_gnavus*	0.016	0.25	7.18E-04	3.32E-03	2.21
		*Ruminococcaceae*	*Fournierella*	0.023	0.33	2.65E-04	1.03E-04	-1.36
*Erysipelotrichia*	*Erysipelotrichales*	*Erysipelotrichaceae*	*Catenibacterium*	0.004	0.12	2.53E-03	2.68E-03	0.08
*Negativicutes*	*Selenomonadales*	*Acidaminococcaceae*	*Succiniclasticum*	0.046	0.38	1.37E-05	1.67E-04	3.61
		*Veillonellaceae*	*Veillonella*	0.029	0.36	3.30E-03	2.33E-03	-0.50
***Proteobacteria***								
*Gammaproteobacteria*	*Pasteurellales*	*Pasteurellaceae*	*Actinobacillus*	0.043	0.39	0.00	1.08E-05	
* *	*Pseudomonadales*	*Moraxellaceae*	*Acinetobacter*	0.043	0.35	7.29E-07	0.00	

Differential abundance analysis was performed using Wilcoxon rank-sum test at genus level.

False discovery rate was controlled based on Benjamini-Hochberg procedure.

### Microbes associated with CAD development

Univariate analysis was performed to investigate the effects of variables on CAD development. Dyslipidemia, diabetes mellitus, lower abundance of *Lachnospiraceae NK4B4* group, *Lachnospiraceae UCG-004*, and *Ruminococcus Gauvreauii*, and higher abundance of *Ruminococcus Gnavus* were significantly associated with CAD (Table 3). The lower relative abundance of *Ruminococcus Gauvreauii* and the higher relative abundance of *Ruminococcus Gnavus* were significantly associated with CAD after adjustment for diabetes mellitus and dyslipidemia (multivariate set 1; *P* = 0.007 and *P* = 0.004, respectively) and after adjustment for diabetes mellitus, dyslipidemia, and the use of aspirin and long-acting nitrate (multivariate set 2; P = 0.011 and 0.004, respectively). The lower relative abundance of *Lachnospiraceae NK4B4*showed a strong trend with the presence of advanced CAD in multivariate set 1 (*P* = 0.063) (Table 4).

**Table 3:** Univariate logistic regression analysis to predict the development of CAD.

	Odds ratio	95% CI	*P* value
Hypertension	0.93	[0.43–1.99]	0.85
Dyslipidemia	3.39	[1.49–7.72]	0.004
Diabetes Mellitus	6.59	[1.78–24.37]	0.005
Chronic kidney disease	1.00	[0.32–3.09]	1.00
Smoking history	1.46	[0.68–3.14]	0.33
*Lachnospiraceae Anaerosporobacter* *	0.15	[0.02–1.33]	0.088
*Lachnospiraceae NK4B4* group*	0.43	[0.21–0.88]	0.015
*Lachnospiraceae UCG-004* *	0.25	[0.08–0.82]	0.012
*Ruminococcus Gauvreauii* group*	0.27	[0.12–0.59]	0.001
Ruminococcus Gnavus*	2.20	[1.22–3.99]	0.006

*1 increase in Log_10_ (relative abundance × 10^5^)

**Table 4:** Multivariate logistic regression analysis to predict the development of CAD.

	Multivariate 1			Multivariate 2		
	Adjusted odds ratio	95% CI	*P* value	Adjusted odds ratio	95% CI	*P* value
*Anaerosporobacter (Lachnospiraceae)*	0.24	[0.04–1.67]	0.15	0.06	[7.88E-19-3.86E15]	0.19
*NK4B4 group (Lachnospiraceae)*	0.16	[0.02–1.16]	0.063	0.59	[0.26–1.34]	0.20
*UCG-004 (Lachnospiraceae)*	0.37	[0.10–1.30]	0.10	0.44	[0.12–1.64]	0.21
*Gauvreauii group (Ruminococcus)*	0.03	[0.002–0.55]	0.007	0.32	[0.12–0.86]	0.011
*Gnavus (Ruminococcus)*	2.72	[1.28–5.79]	0.004	3.01	[1.32–6.87]	0.004

1 increase in Log_10_ (relative abundance × 10^5^).

Multivariate 1; adjusted for dyslipidemia and diabetes mellitus.

Multivariate 2; adjusted for dyslipidemia, diabetes mellitus, aspirin, and long-acting nitrate.

Finally, sensitivity analysis was performed using the whole population. We identified 17 differentially abundant taxa between patients with advanced CAD (N = 96) and patients without CAD (N = 117) in the whole population (S3 Table). In multivariate analysis, two covariate sets were investigated: (1) cardiovascular risk factors which were different between groups (multivariate set 3; age, sex, race, BMI, hypertension, diabetes mellitus, dyslipidemia, and smoking status) and (2) cardiovascular risk factors and medications which were different between groups (multivariate set 4; multivariate set 3, aspirin, long-acting nitrate, and multi-Vitamins). The lower relative abundance of *Ruminococcus Gauvreauii* and the higher relative abundance of *Ruminococcus Gnavus* remained to be significantly associated with CAD in the whole population (S4 Table).

## Discussion

A substantial number of human diseases are associated with compositional alteration of the gut microbiome. Growing evidence suggests that gut microbiome plays a vital role in the development of CAD in human, and several prebiotic/probiotic treatments targeting gut microbiome successfully reduces atherosclerosis in mice. However, the complex interactions between the gut microbiome and hosts as well as numerous confounding factors make it challenging to show the clear association between specific gut microbiomes and the development of CAD in human. The current study demonstrated that advanced CAD patients had distinct microbiome composition compared to age-/sex-/race-/BMI-matched control patients with decreased richness and evenness of the gut microbiome. CAD patients also showed increased relative abundance of *Ruminococcus gnavus* and decreased relative abundance of *Lachnospiraceae NK4B4* and *Ruminococcus gauvreauii* group even after adjustment for dyslipidemia and diabetes mellitus. The putative roles of specific microbes to promote coronary atherosclerosis are described below.

The increased relative abundance of *Ruminococcus gnavus* has been linked to inflammatory bowel disease (IBD) [16–18]. Whether increased abundance of *Ruminococcus gnavus* in IBD is a cause or effect of inflammation is not clear; however, the fact that fecal transplant containing *Ruminococcus gnavus* was associated with a higher incidence of relapse of ulcerative colitis suggested the causal role of *Ruminococcus gnavus in IBD* [19]. Other inflammatory diseases, such as spondyloarthritis and eczema, were also associated with *Ruminococcus gnavus* [20] [21]. Recently published data demonstrated that *Ruminococcus Gnavus* produces an inflammatory polysaccharide, a complex glucorhamnan polysaccharide with a rhamnose backbone and glucose sidechains, which induces tumor necrosis factor-alpha (TNF-α) production by dendritic cells through toll-like receptor 4, filling the mechanistic gap between *Ruminococcus gnavus* and inflammatory diseases [22]. TNF-α is a potent pleiotropic cytokine which exerts inflammatory effects in the development of atherosclerosis: (1) TNF-α promotes macrophages to uptake oxidized LDL and to form foam cells, (2) TNF-α promotes leukocyte recruitment by inducing endothelial VCAM-1 expression, (3) TNF-α promotes chemotaxis by increasing the production of MCP-1from the endothelial cells, (4) TNF-α directly impairs nitric oxide bioavailability by inhibiting endothelial nitric oxide synthase and increasing reactive oxygen species [23] [24]. Conversely, anti-TNF-α treatment improves endothelial function and reduces cardiovascular events [25] [26]. We observed the increased relative abundance of *Ruminococcus gnavus* in patients with advanced CAD, and increase of *Ruminococcus gnavus* was a robust predictor of advanced CAD after adjustment for conventional coronary risk factors such as hyperlipidemia and diabetes mellitus. In this context, inflammation might be a mechanistic link between *Ruminococcus gnavus* abundance and CAD development. Another mechanism might be that *Ruminococcus gnavus* can metabolize mucin as a carbon source which could contribute to gut barrier dysfunction and subsequent increase in immune exposure to foreign materials [27]. To the best of our knowledge, this is the first study to report the association between *Ruminococcus gnavus* and the increased risk of CAD.

*Lachnospiraceae* family is abundant in the mammalian digestive tracts. Many species within this family can produce butyrate through the fermentation of dietary fiber [28]. Recent studies suggest that the cardioprotective effects of dietary fiber consumption are mediated by gut microbiome with increased abundance of butyrate-producing microbes [29] [30]. Butyrate has been recognized to have beneficial effects on the hosts, including trophic and anti-inflammatory effects on colonocytes [31] [32]. Our findings that advanced CAD patients had a reduced relative abundance of *Lachnospiraceae NK4B4* and other *Lachnospiraceae* family members suggests a possibility that butyrate depletion could lead to an increase in inflammation.

The other taxon which was less abundant in advanced CAD patients, *Ruminococcus gauvreauii*, mainly produces acetate as an end-product of the fermentation [33]. Acetate supplementation prevented the development of hypertension in mineralocorticoid excess-treated mice and downregulated cardiac *Egr1*, which has been implicated in human atherosclerosis [34] [35]. One recent randomized, open-label controlled trial demonstrated that dietary restriction of advanced glycation end products resulted in the significantly increased relative abundance in *Ruminococus gauvreauii* [36]. Therefore, the lower relative abundance of *Ruminococcus gauvreauii* in CAD patients in our study might be the consequence of greater prevalence of diabetes mellitus or higher dietary intake of advanced glycation end products which has been linked to increased risk of CAD [37].

The present study has some limitations. First, the gut microbiome was analyzed by 16S rDNA sequencing analysis, which has low phylogenic power at the species level. Second, we did not perform coronary angiography for all the participants; thus, there might have been subclinical CAD in some controls. Third, a relatively small number of patients were included in the final microbiome analysis to minimize the effects of age, sex, race, and BMI on microbiome composition. Fourth, the comparison of serum levels of short-chain free fatty acids and inflammatory markers between CAD patients and controls might have strengthened our findings, but we could not address it because of the lacking of samples. The causal link between the specific microbes and CAD was beyond the scope of this paper. However, microbiome-producing short-chain free fatty acids including butyrate and acetate, inflammation, and dietary pattern might be the potential mechanisms of protecting against or promoting CAD through the alteration of gut microbiome based on the previous literature. Therefore, further studies are necessary to evaluate the causal relationship.

## Conclusion

This study demonstrates that advanced CAD patients have a distinct gut microbiome compared to control patients, with reduced complement of specific butyrate- and acetate-producing gut microbes and increased relative abundance of specific inflammatory polysaccharide- producing gut microbe in advanced CAD patients compared to age-/sex-/race-/BMI-matched controls. Our findings raise the possibility that gut microbiome may be a valuable target for preventing CAD. The mechanism underlying the association between these microbes and CAD needs to be defined in future studies.

## Supporting information

S1 Table Baseline characteristics comparing patients with advanced CAD vs. those without advanced CAD in the whole population. CAD, coronary artery disease; LDL-C, low-density lipoprotein cholesterol; HDL-C, high-density lipoprotein cholesterol; FPG, fasting plasma glucose; BMI, body mass index; BP, blood pressure; O.T.U., operational taxonomic units; F/B ratio, *Fermicutes* to *Bacteroidetes* ratio.
(DOCX)

S2 Table Baseline characteristics comparing patients included vs. excluded in the analysis. CAD, coronary artery disease; LDL-C, low-density lipoprotein cholesterol; HDL-C, high-density lipoprotein cholesterol; FPG, fasting plasma glucose; BMI, body mass index; BP, blood pressure.
(DOCX)

S3 Table Differentially abundant taxa between patients with and without advanced CAD samples at genus level in the whole population. Differential abundance analysis was performed using Wilcoxon rank-sum test at genus level. False discovery rate was controlled based on Benjamini-Hochberg procedure.
(DOCX)

S4 Table Multivariate logistic regression analysis to predict the development of CAD in the whole population. 1 increase in Log_10_ (relative abundance × 10^5^). Multivariate 3; adjusted for age, sex, race, body mass index, hypertension, dyslipidemia, diabetes mellitus, and smoking status. Multivariate 4; adjusted for age, sex, race, body mass index, hypertension, dyslipidemia, diabetes mellitus, smoking status, aspirin, long-acting nitrate, and multi-Vitamins.
(DOCX)
