# Supplementary material for: Coronary artery disease is associated with an altered gut microbiome composition
Source: PLoS One. 2020 Jan 29;15(1):e0227147. doi: 10.1371/journal.pone.0227147 (PMC6988937; doi:10.1371/journal.pone.0227147)
Supplement: S1 Table — CAD, coronary artery disease; LDL-C, low-density lipoprotein cholesterol; HDL-C, high-density lipoprotein cholesterol; FPG, fasting plasma glucose; BMI, body mass index; BP, blood pressure; O.T.U., operational taxonomic units; F/B ratio, Fermicutes to Bacteroidetes ratio. (DOCX) [file pone.0227147.s001.docx]

**S1 Table. Baseline characteristics comparing patients with advanced CAD vs. those without advanced CAD in the whole population**

|  | **Advanced CAD - patients** | **Advanced CAD + patients** | ***P* Value** |
| --- | --- | --- | --- |
|  | **N=117** | **N=96** |  |
| Age, years | 48.8±16.6 | 65.7±9.4 | <0.0001 |
| Sex, n (%) |  |  |  |
| Male | 48 (41.0) | 74 (77.1) | <0.0001 |
| Female | 69 (59.0) | 22 (22.9) |  |
| Race, n (%) |  |  |  |
| Caucasian | 112 (95.7) | 96 (100.0) | 0.040 |
| Non-Caucasian | 5 (4.3) | 0 (0.0) |  |
| Comorbidities, n (%) |  |  |  |
| Hypertension | 31 (26.5) | 54 (56.3) | <0.0001 |
| Diabetes Mellitus | 3 (2.6) | 26 (27.1) | <0.0001 |
| Dyslipidemia | 29 (25.2) | 65 (67.7) | <0.0001 |
| Chronic kidney disease | 10 (8.7) | 14 (14.6) | 0.18 |
| Coronary artery disease | 0 (0.0) | 96 (100.0) | <0.0001 |
| Smoking |  |  |  |
| Current | 7 (6.0) | 7 (7.3) | 0.007 |
| Former | 34 (29.1) | 47 (49.0) |  |
| Never | 76 (65.0) | 42 (43.8) |  |
| Laboratory data |  |  |  |
| LDL-C, mg/dL | 108.8±65.0 | 92.9±38.8 | 0.011 |
| HDL-C, mg/dL | 58 (49-74) | 44 (36-55) | <0.0001 |
| Triglyceride, mg/dL | 93 (66-131) | 120 (91-180) | 0.0002 |
| FPG, mg/dL | 90 (84-97) | 108 (101-139) | <0.001 |
| Creatinine, mg/dL | 0.91±0.19 | 1.02±0.22 | 0.001 |
| BMI, kg/m^2^ | 27.5±5.8 | 30.9±5.9 | <0.0001 |
| Systolic BP, mmHg | 119.2±16.7 | 129.9±19.1 | <0.0001 |
| Diastolic BP, mmHg | 73.7±9.5 | 72.6±10.6 | 0.46 |
| Medications, n (%) |  |  |  |
| Aspirin | 40 (34.2) | 80 (83.3) | <0.0001 |
| Statin | 26 (22.2) | 70 (72.9) | <0.0001 |
| Long-acting nitrate | 10 (8.6) | 48 (50.0) | <0.0001 |
| Antihypertensive | 44 (37.6) | 76 (79.2) | <0.0001 |
| Antidiabetic | 5 (4.3) | 24 (25.0) | <0.0001 |
| Proton-pump inhibitor | 16 (13.7) | 22 (22.9) | 0.080 |
| Multi-vitamins | 49 (41.9) | 27 (28.1) | 0.037 |
| Alcohol consumption, drinks/week | 1 (0-3) | 2 (0-2) | 0.88 |
| α-diversity |  |  |  |
| Chao-1 | 955.8±213.8 | 795.0±168.9 | <0.0001 |
| Shannon index | 6.2 (5.8-6.6) | 5.9 (5.4-6.2) | <0.0001 |
| O.T.U. | 766.8±187.8 | 621.3±140.2 | <0.0001 |
| F/B ratio | 2.10 (1.49-4.96) | 3.24 (1.55-8.53) | 0.026 |

CAD, coronary artery disease; LDL-C, low-density lipoprotein cholesterol; HDL-C, high-density lipoprotein cholesterol; FPG, fasting plasma glucose; BMI, body mass index; BP, blood pressure; O.T.U., operational taxonomic units; F/B ratio, *Fermicutes* to *Bacteroidetes* ratio.
