# Supplementary material for: Coronary artery disease is associated with an altered gut microbiome composition
Source: PLoS One. 2020 Jan 29;15(1):e0227147. doi: 10.1371/journal.pone.0227147 (PMC6988937; doi:10.1371/journal.pone.0227147)
Supplement: S2 Table — CAD, coronary artery disease; LDL-C, low-density lipoprotein cholesterol; HDL-C, high-density lipoprotein cholesterol; FPG, fasting plasma glucose; BMI, body mass index; BP, blood pressure. (DOCX) [file pone.0227147.s002.docx]

**S2 Table. Baseline characteristics comparing patients included vs. excluded in the analysis**

|  | Total N=213 |  | Excluded patients N=107 | Included patients N=106 | *P* Value |
| --- | --- | --- | --- | --- | --- |
| Age, years | 56.4±16.2 |  | 50.0±18.8 | 62.8±9.4 | <0.0001 |
| Sex, n (%) |  |  |  |  |  |
| Male | 122 (57.3) |  | 60 (56.1) | 62 (58.5) | 0.72 |
| Female | 91 (42.7) |  | 47 (43.9) | 44 (41.5) |  |
| Race, n (%) |  |  |  |  |  |
| Caucasian | 208 (97.7) |  | 102 (95.3) | 106 (100.0) | 0.024 |
| Non-Caucasian | 5 (2.3) |  | 5 (4.7) | 0 (0.0) |  |
| Comorbidities, n (%) |  |  |  |  |  |
| Hypertension | 85 (39.9) |  | 34 (31.8) | 51 (48.1) | 0.015 |
| Diabetes Mellitus | 29 (13.6) |  | 11 (10.3) | 18 (17.0) | 0.15 |
| Dyslipidemia | 94 (44.6) |  | 32 (29.9) | 62 (59.6) | <0.0001 |
| Chronic kidney disease | 24 (11.3) |  | 10 (9.5) | 14 (13.2) | 0.40 |
| Coronary artery disease | 96 (45.1) |  | 43 (40.2) | 53 (50.0) | 0.15 |
| Smoking |  |  |  |  |  |
| Current | 14 (6.6) |  | 5 (4.7) | 9 (8.5) | 0.41 |
| Former | 81 (38.0) |  | 39 (36.5) | 42 (39.6) |  |
| Never | 118 (55.4) |  | 63 (58.9) | 55 (51.9) |  |
| Laboratory data |  |  |  |  |  |
| LDL-C, mg/dL | 103±37 |  | 100.6±34.0 | 105.9±41.0 | 0.38 |
| HDL-C, mg/dL | 53 (43-68) |  | 55 (44-74) | 50 (42-62) | 0.19 |
| Triglyceride, mg/dL | 102 (78-144) |  | 94 (70-141) | 118 (87-154) | 0.038 |
| FPG, mg/dL | 96 (88-108) |  | 91 (85-105) | 101 (93-119) | 0.001 |
| Creatinine, mg/dL | 0.96±0.21 |  | 0.94±0.21 | 0.98±0.21 | 0.19 |
| BMI, kg/m^2^ | 29.0±6.1 |  | 28.8±6.4 | 29.2±5.8 | 0.57 |
| Systolic BP, mmHg | 124.0±18.6 |  | 120.3±17.7 | 127.9±18.7 | 0.003 |
| Diastolic BP, mmHg | 73.2±10.0 |  | 73.1±9.7 | 73.3±10.4 | 0.87 |
| Medications, n (%) |  |  |  |  |  |
| Aspirin | 120 (56.3) |  | 47 (43.9) | 73 (68.9) | 0.0002 |
| Statin | 96 (45.1) |  | 36 (33.6) | 60 (56.6) | 0.0008 |
| Long-acting nitrate | 58 (27.2) |  | 28 (26.2) | 30 (28.3) | 0.73 |
| Antihypertensive | 120 (56.3) |  | 46 (43.0) | 74 (69.8) | <0.0001 |
| Antidiabetic | 29 (13.6) |  | 13 (12.2) | 16 (15.1) | 0.53 |
| Proton-pump inhibitors | 38 (17.8) |  | 18 (16.8) | 20 (18.9) | 0.70 |
| Multi-vitamins | 76 (35.7) |  | 40 (37.4) | 36 (34.0) | 0.60 |
| Alcohol consumption, drinks/week | 1 (0-3) |  | 1 (0.25-3) | 1.25 (0-3) | 0.63 |

LDL-C, low density lipoprotein cholesterol; HDL-C, high density lipoprotein cholesterol; FPG, fasting plasma glucose; BMI, body mass index; BP, blood pressure.
