# Supplementary material for: Coronary artery disease is associated with an altered gut microbiome composition
Source: PLoS One. 2020 Jan 29;15(1):e0227147. doi: 10.1371/journal.pone.0227147 (PMC6988937; doi:10.1371/journal.pone.0227147)
Supplement: S3 Table — Differential abundance analysis was performed using Wilcoxon rank-sum test at genus level. False discovery rate was controlled based on Benjamini-Hochberg procedure. (DOCX) [file pone.0227147.s003.docx]

**S3 Table.** **Differentially abundant taxa between patients with and without advanced CAD samples at genus level in the whole population**

| ***Phylum/***  ***Class*** | ***Order*** | ***Family*** | ***Genus*** | ***P* value** | ***Q* value** | **Advanced CAD - (N=117)**  **Mean** | **Advanced CAD + (N=96)**  **Mean** | **Log2 fold change** |
| --- | --- | --- | --- | --- | --- | --- | --- | --- |
| ***Actinobacteria*** |  |  |  |  |  |  |  |  |
| *Coriobacteriia* | *Coriobacteriales* | *Atopobiaceae* | *Olsenella* | 0.007 | 0.064 | 2.12E-04 | 2.70E-04 | 0.35 |
|  |  | *Coriobacteriaceae* | *Enorma* | <0.0001 | 0.002 | 8.41E-05 | 8.78E-05 | 0.06 |
|  |  | *Eggerthellaceae* | *Gordonibacter* | 0.006 | 0.058 | 7.39E-04 | 8.39E-04 | 0.18 |
| ***Bacteroidetes*** |  |  |  |  |  |  |  |  |
| *Bacteroidia* | *Bacteroidales* | *Barnesiellaceae* | *Coprobacter* | 0.044 | 0.206 | 5.98E-04 | 1.76E-04 | -1.76 |
|  |  | *Porphyromonadaceae* | *Porphyromonas* | 0.014 | 0.093 | 1.22E-04 | 9.55E-05 | -0.35 |
|  |  | *Prevotellaceae* | *Alloprevotella* | 0.0008 | 0.012 | 1.73E-03 | 4.66E-04 | -1.89 |
|  |  |  | *Paraprevotella* | 0.0005 | 0.008 | 1.87E-03 | 3.57E-03 | 0.93 |
|  |  |  | *Prevotella* | 0.008 | 0.069 | 3.38E-04 | 1.63E-04 | -1.05 |
|  |  |  | *Prevotella_7* | 0.044 | 0.200 | 1.14E-03 | 2.47E-03 | 1.12 |
| ***Firmicutes*** |  |  |  |  |  |  |  |  |
| *Clostridia* | *Clostridiales* | *Defluviitaleaceae* | *Defluviitaleaceae_UCG-011* | 0.015 | 0.096 | 1.48E-04 | 7.59E-05 | -0.96 |
|  |  | *Family_XIII* | *Family_XIII_UCG-001* | 0.047 | 0.198 | 3.33E-04 | 2.12E-04 | -0.65 |
|  |  |  | *Mogibacterium* | 0.008 | 0.066 | 1.85E-04 | 3.61E-04 | 0.96 |
|  |  |  | *[Eubacterium]_brachy_group* | 0.029 | 0.148 | 1.94E-04 | 3.42E-04 | 0.82 |
|  |  | *Lachnospiraceae* | *Agathobacter* | <0.0001 | 0.002 | 1.55E-02 | 2.48E-02 | 0.68 |
|  |  |  | *Anaerosporobacter* | <0.0001 | 0.017 | 1.63E-02 | 2.58E-05 | -9.30 |
|  |  |  | *Coprococcus_3* | 0.002 | 0.020 | 3.20E-03 | 2.01E-03 | -0.67 |
|  |  |  | *Eisenbergiella* | <0.0001 | 0.002 | 8.60E-04 | 8.58E-04 | -0.003 |
|  |  |  | *Fusicatenibacter* | 0.008 | 0.063 | 1.23E-02 | 7.27E-03 | -0.76 |
|  |  |  | *Howardella* | 0.0008 | 0.011 | 1.15E-04 | 1.71E-04 | 0.57 |
|  |  |  | *Lachnospiraceae_NK4B4* | <0.0001 | 0.003 | 4.48E-04 | 1.82E-04 | -1.30 |
|  |  |  | *Lachnospiraceae_UCG-004* | <0.0001 | 0.003 | 2.29E-04 | 3.45E-05 | -2.73 |
|  |  |  | *Lachnospiraceae_UCG-010* | 0.034 | 0.168 | 3.75E-04 | 1.81E-04 | -1.05 |
|  |  |  | *Lactonifactor* | 0.011 | 0.076 | 1.65E-03 | 2.38E-03 | 0.53 |
|  |  |  | *Marvinbryantia* | 0.001 | 0.012 | 5.08E-04 | 6.36E-04 | 0.32 |
|  |  |  | *Sellimonas* | 0.034 | 0.168 | 3.36E-04 | 6.10E-04 | 0.86 |
|  |  |  | *Tyzzerella* | 0.045 | 0.200 | 8.69E-04 | 5.59E-04 | -0.64 |
|  |  |  | *Tyzzerella_4* | <0.0001 | 0.002 | 1.18E-03 | 1.71E-03 | 0.54 |
|  |  |  | *[Ruminococcus]_gauvreauii* | <0.0001 | 0.006 | 1.36E-03 | 1.02E-04 | -3.74 |
|  |  |  | *[Ruminococcus]_gnavus* | 0.0009 | 0.011 | 4.83E-03 | 2.23E-03 | -1.11 |
|  |  | *Peptostreptococcaceae* | *Romboutsia* | 0.018 | 0.104 | 1.68E-02 | 1.08E-02 | -0.64 |
|  |  | *Ruminococcaceae* | *CAG-352* | 0.047 | 0.203 | 2.40E-04 | 6.07E-04 | 1.34 |
|  |  |  | *Faecalibacterium* | 0.038 | 0.183 | 3.55E-02 | 2.59E-02 | -0.45 |
|  |  |  | *Flavonifractor* | 0.015 | 0.093 | 2.92E-03 | 2.10E-03 | -0.48 |
|  |  |  | *Fournierella* | <0.0001 | 0.004 | 8.03E-04 | 6.99E-05 | -3.52 |
|  |  |  | *Oscillibacter* | 0.048 | 0.198 | 3.35E-03 | 2.13E-03 | -0.65 |
| *Erysipelotrichia* | *Erysipelotrichales* | *Erysipelotrichaceae* | *Coprobacillus* | 0.0012 | 0.013 | 3.12E-04 | 1.48E-04 | -1.08 |
|  |  |  | *Dielma* | 0.027 | 0.146 | 3.53E-04 | 1.63E-04 | -1.11 |
|  |  |  | *Holdemania* | 0.016 | 0.095 | 6.92E-04 | 5.11E-04 | -0.44 |
| *Negativicutes* | *Selenomonadales* | *Acidaminococcaceae* | *Succiniclasticum* | 0.0097 | 0.073 | 5.88E-05 | 6.50E-04 | 3.47 |
|  |  | *Veillonellaceae* | *Veillonella* | 0.0098 | 0.071 | 1.69E-03 | 1.53E-03 | -0.14 |
| ***Proteobacteria*** |  |  |  |  |  |  |  |  |
| *Gammaproteobacteria* | *Betaproteobacteriales* | *Burkholderiaceae* | *Parasutterella* | 0.022 | 0.123 | 2.30E-03 | 1.70E-03 | -0.44 |
|  | *Enterobacteriales* | *Enterobacteriaceae* | *Escherichia-Shigella* | 0.028 | 0.147 | 1.32E-02 | 2.93E-02 | 1.15 |
|  |  | *Enterobacteriaceae* | *Klebsiella* | <0.0001 | 0.009 | 3.69E-04 | 6.64E-03 | 4.17 |

Differential abundance analysis was performed using Wilcoxon rank-sum test at genus level.

False discovery rate was controlled based on Benjamini-Hochberg procedure.
