# Supplementary material for: Coronary artery disease is associated with an altered gut microbiome composition
Source: PLoS One. 2020 Jan 29;15(1):e0227147. doi: 10.1371/journal.pone.0227147 (PMC6988937; doi:10.1371/journal.pone.0227147)
Supplement: S4 Table — 1 increase in Log10 (relative abundance × 105). Multivariate 3; adjusted for age, sex, race, body mass index, hypertension, dyslipidemia, diabetes mellitus, and smoking status. Multivariate 4; adjusted for age, sex, race, body mass index, hypertension, dyslipidemia, diabetes mellitus, smoking status, aspirin, long-acting nitrate, and multi-Vitamins. (DOCX) [file pone.0227147.s004.docx]

**S4 Table. Multivariate logistic regression analysis to predict the development of CAD in the whole population**

|  | Multivariate 3 | | | Multivariate 4 | | |
| --- | --- | --- | --- | --- | --- | --- |
|  | Adjusted odds ratio | 95% CI | *P* value | Adjusted odds ratio | 95% CI | *P* value |
| *Enorma (Coriobacteriaceae)* | 0.05 | [0-.] | 0.99 | 7.85E-04 | [0-.] | 0.99 |
| *Alloprevotella (Prevotellaceae)* | 1.22E-05 | [0-.] | 1.00 | 1.69E+07 | [0-.] | 1.00 |
| *Paraprevotella (Prevotellaceae)* | 0.47 | [0.07-3.14] | 0.43 | 0.77 | [0.44-1.34] | 0.35 |
| *Agathobacter (Lachnospiraceae)* | 0.74 | [0.39-1.41] | 0.35 | 0.77 | [0.36-1.63] | 0.50 |
| *Anaerosporobacter (Lachnospiraceae)* | 0.12 | [9.38E-03-1.51] | 0.10 | 5.97E+03 | [0-.] | 1.00 |
| *Coprococcus_3 (Lachnospiraceae)* | 0.61 | [0.37-1.00] | 0.051 | 0.50 | [0.27-0.90] | 0.022 |
| *Eisenbergiella (Lachnospiraceae)* | 1.63 | [0.91-2.92] | 0.10 | 1.81 | [0.95-3.47] | 0.073 |
| *Howardella (Lachnospiraceae)* | 0.75 | [0.30-1.89] | 0.54 | 0.56 | [0.17-1.88] | 0.35 |
| *NK4B4 (Lachnospiraceae)* | 0.49 | [0.23-1.02] | 0.053 | 0.55 | [0.24-1.25] | 0.15 |
| *UCG-004 (Lachnospiraceae)* | 0.18 | [0.05-0.63] | 0.003 | 0.18 | [0.04-0.78] | 0.022 |
| *Marvinbryantia (Lachnospiraceae)* | 1.08 | [0.53-2.18] | 0.84 | 0.95 | [0.43-2.08] | 0.89 |
| *Tyzzerella_4 (Lachnospiraceae)* | 1.73 | [1.03-2.89] | 0.037 | 1.52 | [0.85-2.73] | 0.16 |
| *Gauvreauii (Ruminococcaceae)* | 0.22 | [0.10-0.50] | 0.0003 | 0.26 | [0.11-0.62] | 0.003 |
| *Gnavus (Ruminococcaceae)* | 2.37 | [1.26-4.46] | 0.007 | 2.90 | [1.39-6.08] | 0.005 |
| *Fournierella (Ruminococcaceae)* | 0.89 | [0.41-1.92] | 0.76 | 1.38 | [0.58-3.27] | 0.46 |
| *Coprobacillus (Erysipelotrichaceae)* | 1.29 | [0.60-2.76] | 0.51 | 1.59 | [0.69-3.69] | 0.28 |
| *Klebsiella (Erysipelotrichaceae)* | 0.99 | [0.60-1.65] | 0.98 | 0.96 | [0.53-1.73] | 0.89 |

1 increase in Log_10_ (relative abundance × 10^5^).

Multivariate 3; adjusted for age, sex, race, body mass index, hypertension, dyslipidemia, diabetes mellitus, and smoking status.

Multivariate 4; adjusted for age, sex, race, body mass index, hypertension, dyslipidemia, diabetes mellitus, smoking status, aspirin, long-acting nitrate, and multi-Vitamins.
